# Investigation of Cellular Interactions of Lipid-Structured Nanoparticles With Oral Mucosal Epithelial Cells

**DOI:** 10.3389/fmolb.2022.917921

**Published:** 2022-05-23

**Authors:** R. Jeitler, C. Glader, C. Tetyczka, S. Zeiringer, M. Absenger-Novak, A. Selmani, E. Fröhlich, E. Roblegg

**Affiliations:** ^1^ Pharmaceutical Technology and Biopharmacy, Institute of Pharmaceutical Sciences, University of Graz, Universitätsplatz 1, Graz, Austria; ^2^ Research Center Pharmaceutical Engineering GmbH, Graz, Austria; ^3^ Center for Medical Research, Medical University of Graz, Graz, Austria

**Keywords:** lipid-structured nanoparticles, oral mucosa, cellular interaction and uptake, solid lipid nanoparticles, nanostructured lipid carriers, matrix composition, design of experiments (DoE), endocytic uptake mechamism

## Abstract

Lipid-based nanosystems enable intracellular delivery of drugs in the oral cavity for the treatment of local diseases. To rationally design such systems, suitable matrix compositions and particle properties need to be identified, and manufacturing technologies that allow reproducible production have to be applied. This is a prerequisite for the reliable and predictable performance of *in-vitro* biological studies. Here, we showed that solid lipid nanoparticles (SLN, palmitic acid) and nanostructured lipid carriers (NLC, palmitic acid and oleic acid in different ratios) with a size of 250 nm, a negative zeta potential, and a polydispersity index (PdI) of less than 0.3 can be reproducibly prepared by high-pressure homogenization using quality by design and a predictive model. SLN and NLC were colloidally stable after contact with physiological fluid and did not form agglomerates. The *in-vitro* studies clearly showed that besides particle size, surface charge and hydrophobicity, matrix composition had a significant effect. More specifically, the addition of the liquid lipid oleic acid increased the cellular uptake capacity without changing the underlying uptake mechanism. Regardless of the matrix composition, caveolin-mediated endocytosis was the major route of uptake, which was confirmed by particle localization in the endoplasmic reticulum. Thus, this work provides useful insights into the optimal composition of lipid carrier systems to enhance the intracellular uptake capacity of drugs into the oral mucosa.

## 1 Introduction

Drug administration via the oral mucosa is considered a potential route as it provides several benefits compared to traditional administration. In particular, oral diseases such as oral cancer, ulcers, mucositis, or *candida* can be treated locally, which reduces dose and thus limits side effects. However, regarding local drug delivery it must be taken into account that the mouth is a well-organized and complex system, which has a variety of functions that might influence drug transport. Among these, the mouth represents the first line of defense against undesirable substances, viruses and bacteria and prevents their penetration. This is due to the fact, that the anatomy and physiology of the oral mucosa is very complex. (Sankar et al., 2011; Squier and Brodgen, 2011). It comprises keratinized and non-keratinized regions that are permanently flushed with saliva to keep the environment moist and foster the growth of characteristic microorganisms. (Roblegg, Coughran and Sirjani, 2019). Thereby, drugs more easily penetrate the non-keratinized regions, which include the buccal and sublingual areas, than the keratinized regions, such as the gingiva. Hence, absorption of drugs is often difficult and depends strongly on the physicochemical properties including solubility, lipophilicity, molecular weight and others. (Lam et al., 2020). Nanoparticles (NP) are one strategy for improving the delivery of e.g., poorly soluble or enzymatically degradable drugs into the oral mucosa. Their small sizes facilitate intracellular delivery of potential therapeutic candidates to the site of action. Like a double-edged sword, this ability also gives rise to cytotoxic effects. (Foroozandeh and Aziz, 2018). Thus, the safe and effective penetration into cells and the intracellular fate of NP is crucial for the performance. This still remains challenging to control, as NP development is often hampered by a lack of understanding of how, for example, the composition of the nano-scaled carrier affects the interaction with the oral epithelium including saliva. (Behzadi et al., 2017).

Recent studies have shown that size and surface functionalization of NP have a significant influence on the colloidal stability in saliva and, accordingly, on cellular interactions. The microstructure of saliva in principle favors the adsorption of NP. (Teubl et al., 2018). It was found that a positive surface charge led to agglomeration due to interactions with polyvalent ions in saliva, while negatively and neutrally charged polymer NP were only moderately affected. These effects were generally more pronounced for larger particles (200 nm) than for smaller ones (20 nm) because of their lower surface curvature. Moreover, non-functionalized NP, similar to viruses, did not interact with the salivary mucoglycoproteins in contrast to their functionalized counterparts, which were increasingly immobilized during transit. Once NP have crossed the salivary pellicle, they reach the underlying epithelium. It is important to note that the surface membrane of the epithelium is covered by ridge-like folds, also known as microplicae. (Kullaa-Mikkonen, 1987; Gao, Shi and Freund, 2005). These microplicae show diameters ranging from 200 to 500 nm. Hence, NP that perfectly fit into these folds, i.e., about 200–400 nm deposit there, immediately get in contact with the cell membrane and endocytotic uptake is driven by thermodynamic driving forces and/or less inter-particle electric repulsion compared to smaller particles. (Teubl, Meindl, et al., 2013). An alternative to polymer NP are lipid-based nanosystems. According to Garcia-Pinel et al., classic representatives of this class are liposomes, solid lipid nanoparticles (SLN), and nanostructured lipid carriers (NLC) (García-Pinel et al., 2019). Qi et al. also list lipid-drug conjugates (LDC) and nanoemulsions (NE) as important examples. (Qi et al., 2017). They all have in common that they are mainly composed of physiological lipid analogues with surfactants as stabilizers. (Qi et al., 2017). More specifically, according to Müller, Mäder and Gohla et al., SLN comprise solid lipids and a stabilizer and are considered highly biocompatible and biodegradable but have a limited drug loading capacity (about 25% with respect to the lipid matrix). (Müller, Mäder and Gohla, 2000). NLC consist in principle of solid lipids and liquid lipids (oils); if the proportion of liquid lipid (oil) is low, they are referred to as imperfect-type NLC. (Wissing, Kayser and Müller, 2004; Guimarães and Ré, 2011). A further increase in the oily phase results in so-called multiple-type NLC. Thereby, the amount of liquid lipid plays an important role, as the solubility of active ingredients is often higher in liquid lipids than in solid ones and the active ingredient loading can thus be increased. (Müller, Radtke and Wissing, 2002b). Although several studies have reported on the preparation, drug incorporation mechanisms, pharmacokinetics, and stability of lipid NP, there is little information on how these structures, depending on their composition (saturated versus unsaturated lipids), interact with physiological fluids and consequently, human cells/tissues. (El-Salamouni et al., 2015; Tetyczka et al., 2017). First studies performed by Tetyczka et al. showed that NLC do not agglomerate when dispersed in saliva most likely due to the hydrophobic particle surface. (Tetyczka et al., 2017). Moreover, uptake into epithelial cells of the buccal mucosa occurred *via* active transport, which does not change even in the inflamed state and thus other physiological conditions present. (Tetyczka et al., 2021).

The aim of this study was to investigate the influence of the composition of SLN and NLC on cellular uptake behavior and possible adverse effects on oral mucosal epithelial cells. For this purpose, four formulations were prepared by high-pressure homogenization (HPH). The process and the formulations were adjusted by Design of Experiments (DoE) to produce almost spherically shaped, negatively charged particles with a size of 200–300 nm that exhibit a narrow particle size distribution (e.g., low PdI, low span-value). Thereby, SLN were based on palmitic acid and the stabilizer Tween 80, and the NLC were composed of palmitic acid and different concentrations of the liquid lipid oleic acid as well as Tween 80. The biological interactions of the lipid particles were tested on human buccal epithelial cells (TR 146 cells), and differences in uptake route, intracellular localization, and possible adverse effects were carefully investigated.

## 2 Materials and Methods

For nanoparticle preparation, Tween 80 was used as stabilizer (purity of 73%, Sigma Aldrich, Munich, Germany) and palmitic acid (assay via GC ≥ 98%, Merck, Darmstadt, Germany) as solid lipid. Oleic acid (Croda Inc., Pennsylvania, United States) was used as liquid lipid to prepare NLC. Ultrapurified water (i.e., Milli-Q®-water (MQ-water); Millipore SAS, Molsheim, France) was used for all experiments.

Human buccal TR 146 cells were obtained from Imperial *Cancer* Research Technology (London, United Kingdom). Dulbecco’s Modified Eagle’s medium (DMEM), phosphate buffered saline (PBS), fetal bovine serum (FBS), penicillin streptomycin, Hank’s Balanced Salt Solution (HBSS), and 0.25% trypsin-ethylenediaminetetraacetic acid (trypsin-EDTA) (Gibco, Life Technologies Corporation, Painsley, United Kingdom) were used for cell culture experiments. Oil red o, Dynasore hydrate (purity of 100%), Chlorpromazine hydrochloride (purity of 98%), Genistein (purity of 99%), 5-(N-Ethyl-N-isopropyl)amiloride (EIPA, purity of 100%), MitoRed (purity of 74%) and non-essential amino acid solution (NEAA) were purchased from Sigma Aldrich (Munich, Germany). Dihydroethidium (DHE, purity of 99%), Alexa Fluor 488 Phalloidin (purity of 99%), ER-Tracker™ Red dye, LysoTracker® Red DND-99 and Hoechst 33,342 were obtained from Thermo Fisher Scientific (Vienna, Austria). Acetonitrile was purchased from VWR (Vienna, Austria). HyClone (GE Healthcare Life Sciences, Logan, United States) was used as serum-free DMEM for all experiments.

### 2.1 SLN and NLC Preparation and Characterization

#### 2.1.1 Differential Scanning Calorimetry (DSC) Measurements

DSC measurements were performed to investigate the thermal behavior of the individual components and the particles (DSC 204F1 Phoenix, Netzsch GmbH, Selb, Germany). Furthermore, the suitable process temperatures were identified and the exact type of NLC was determined. For this purpose, 1–10 mg of each solid lipid and of the air-dried SLN (solid lipid and stabilizer) and NLC (solid:liquid lipid in the ratios 9:1, 8:2, and 7:3 with stabilizer), respectively, were weighed into aluminum crucibles and sealed with punctured lids. (El-Salamouni et al., 2015). Samples were scanned from −20 to 100°C in two heating (i.e., heating rate of 10 K/min) and cooling cycles (i.e., cooling rate of 10 K/min); a nitrogen flow rate of 20 ml/min was applied to purge the cell. An empty aluminum crucible was used as reference. All experiments were performed in triplicate and data were analyzed using Netzsch Proteus Software (Netzsch GmbH, Selb, Germany).

#### 2.1.2 Preparation of SLN and NLC *via* HSM and HPH

For blank NP preparation, the Tween 80 concentration in the aqueous phase was set at 2.5% (w/w) and the total amount of lipids at 10% (w/w). For the production of labeled NP, 2% (w/w) of the lipid phase was replaced by oil red o. To achieve reproducible production of the SLN and NLC particles, the required process parameters were identified using DoE studies utilizing MODDE® software (Version 13.0, MKS Umetrics AB, Malmö, Sweden). The Central Composite Face-Centered (CCF) quadratic experimental design was used and the Multiple Linear Regression (MLR) was selected as fitting method. HSM speed (i.e., 8,000–16,000 rpm) and time (i.e., 15–60 s), HPH pressure (i.e., 250–750 bar) and number of cycles (i.e., 1–10) were defined as DoE inputs. Particle size [i.e., D (0.1), D (0.5) and D (0.9)] was selected as response and was determined immediately after production using laser diffraction (LD, Mastersizer 2000, Malvern Instruments, Malvern, United Kingdom; see 2.1.3). For the statistical analysis the MODDE®software was used and coefficient plots, a summary of fit considering R2 (i.e., coefficient of determination), Q2 (i.e., goodness of prediction), model validity and reproducibility as well as response contour plots were created. Based on the findings of the DoE studies, the aqueous phase was mixed with the lipid phase consisting of the solid lipid (i.e., palmitic acid) or a binary mixture of solid and liquid lipid (i.e., oleic acid in the ratios of 9:1, 8:2 and 7:3) in the first step, using HSM (Ultra Turrax T25 digital equipped with S25N-18G, IKA, Staufen, Germany). The process was performed at 12,000 rpm for 30 s while maintaining the product temperature at 71.5°C. Subsequently, the obtained pre-emulsion was transferred to the HPH (Panda 2K, NS1001L Spezial, GEA Niro Soavi, Lübeck, Germany, equipped with a water bath) preheated to 71.5°C and homogenized with six cycles at 500 bar. Finally, the samples were immediately cooled to 4°C in an ice bath to obtain homogeneous products and prevent undesired gelation.

#### 2.1.3 Characterization of SLN and NLC

The particle size and PdI were measured via laser diffraction (LD; Mastersizer 2000, Malvern Instruments, Malvern, United Kingdom) and dynamic light scattering (DLS; Zeta sizer Nano ZS, Malvern Instruments, Malvern, United Kingdom). For LD measurements approximately 15–50 µl of the samples were added to 18–20 ml dispersant (i.e., MQ-water, refractive index = 1.330) to reach an obscuration of 4–6%. The real refractive index was set to 1.438 (i.e., palmitic acid) (Tetyczka et al., 2017) and the imaginary refractive index to 0.010 for SLN and 0.001 for NLC, respectively. D (0.1), D (0.5), D (0.9) and D (0.99) volume-based values were determined in triplicate at 25°C to detect significant changes in the main particle size and the presence of fines or large particles/agglomerates. The values are the maximum particle diameters (D) below which 10%, 50%, 90% and 99% of the sample volume exists.

DLS measurements were conducted immediately after production but also over a period of 1 month on a weekly basis. For this, 15 ml of all formulations were stored in the refrigerator at 4°C. Hydrodynamic particle sizes (Z-average, nm), PdI and zeta potential values (mV) were measured considering values for the refractive index of 1.440 and 0.010 for palmitic acid. To avoid multiple scattering, samples were diluted 1:10,000 in MQ water (i.e., refractive index = 1.330) and serum-free DMEM (i.e., refractive index = 1.335) prior to 173° back scatter measurements at 25°C. The zeta potential was determined via Laser-Doppler-Micro Electrophorese (ELS) coupled with DLS (Nano ZS Malvern Instruments, Malvern, United Kingdom). Measurements were carried out at 25°C applying a scattering angle of 173° after samples were diluted 1:10,000 with zeta-water (i.e., MQ-water adjusted to a pH of 5.5-6 with a conductivity of 50 μS/cm using 0.9% (v/v) of sodium chloride solution). (Baumgartner et al., 2016). All measurements were performed in triplicate and mean values and respective standard deviations (±SD) are presented.

The shape of SLN and NLC particles was investigated through atomic force microscopy (AFM). For this, a FlexAFM atomic force microscope equipped with an Easyscan two controller (Nanosurf, Liestal, Switzerland) and a C3000 control software under ambient conditions was used. 100 µl of the diluted samples (i.e., 10 µg particles/ml) were placed on a silicon waver material tilted at 45° and dried overnight at room temperature. The waver was rinsed with deionized water, ethanol and dried with nitrogen prior to use. Non-contact (tapping) mode with silicon nitride Tap300Al-G cantilevers (Budgetsensors, Sofia, Bulgaria; with 10 nm radius, 125 µm length and nominal spring constant of 40 N/m) with a nominal resonance frequency of 300 kHz was used for the acquisition with a set point of 60%. All acquired data were processed with open access Gwyddion Data Processing Software (version 2.55). (Nečas and Klapeteek, 2012).

### 2.2 Interaction and Uptake Studies of SLN and NLC

#### 2.2.1 Cell Culture

TR 146 cells were cultured in DMEM supplemented with 10% FBS, 1% penicillin streptomycin and 1% NEAA at 37°C in a humidified atmosphere with 5% CO_2_. Medium was changed three times a week and sub-cultivation of confluent cells was conducted on a weekly basis using trypsin-EDTA. For all experiments, serum-free DMEM without phenol red, supplemented with 1% penicillin streptomycin and 1% NEAA was used.

#### 2.2.2 *In-vitro* Cytotoxicity Studies

To elucidate potential cytotoxic effects of SLN and NLC formulations, MTS and LDH assays were performed. For this, TR 146 cells were seeded in 96-well plates (Greiner Bio-One GmbH, Frickenhausen, Germany) using a seeding density of 2 × 10^4^ cells/well and cultured for 24 h. After washing the cells with PBS, the cells were incubated with various SLN and NLC concentrations (i.e., 500 and 750 μg/ml related to the particle concentration) in serum-free DMEM at various concentrations at 37°C for 4 h (*n* = 6). Changes in cell viability were evaluated by a CellTiter 96 Aqueous Non-Radioactive Cell Proliferation Assay (MTS, Promega Corporation, Madison, United States) according to the manufacturer’s instructions. Absorbance was measured at 490 nm after 3 h of incubation using a microplate reader (CLARIOstar^Plus^, BMG LABTECH, Ortenberg, Germany). To calculate the percentage of cell viability untreated blank corrected wells were used as control representing 100% cell viability. Cell membrane integrity was tested using CytoTox-ONE Homogeneous Membrane Integrity Assay (LDH, Promega), which determines the LDH release of the cells after 4 h incubation with labeled SLN and NLC formulations (i.e., 500 and 750 μg/ml related to the particle concentration, *n* = 6) according to the manufacturer’s instructions. Fluorescence measurements were examined at an excitation wavelength of 544 nm and an emission wavelength of 590 nm with an UV-Vis Plate reader (CLARIOstar^Plus^, BMG LABTECH, Ortenberg, Germany). 100% LDH release was triggered by adding 2% of a lysis solution to untreated wells and served as positive control. In addition, all data were blank corrected.

For the evaluation of reactive oxygen species (ROS) production, TR 146 cells were seeded in 96-well plates (NuncTM MicroWellTM 96-Well Optical-Bottom Plates with Polymer Base; Thermo Fisher Scientific) using a seeding density of 6 × 10^4^ cells/well. After 24 h of cultivation, cells were washed with PBS and incubated with a mixture of 10 µM DHE and either SLN or NLC-dispersions (i.e., 500 and 750 μg/ml related to nanoparticle concentration in serum-free DMEM) (n = 6). ROS production was quantified after 4 h by measuring the fluorescence at an excitation wavelength of 544 nm and an emission wavelength of 612 nm using CLARIOstar^Plus^ (BMG LABTECH, Ortenberg, Germany).

#### 2.2.3 Uptake Studies

TR 146 cells were seeded in 8-well chamber glass slides (Falcon®, Corning, Arizona, United States) using a seeding density of 1.5 × 10^4^ cells/well and cultured for 1 week. To carefully assess whether active or passive transport mechanisms are involved in particle uptake, cells were incubated for 4 h with oil red o-labeled NP diluted in serum-free DMEM at a final concentration of 500 and 750 μg/ml (related to the particle concentration) at 37°C (i.e., active transport, *n* = 6) and 4°C (i.e., passive transport, *n* = 6). Untreated cells incubated at 37°C or 4°C for 4 h were used as control for the uptake studies (*n* = 6). For semi-quantitative analysis TR 146 cells were seeded in 24-well polystyrene plates (Falcon, Corning, United States) using a seeding density of 4 × 10^4^ cells/well and cultured for 1 week. Subsequently, the cells were incubated with oil red o labeled NP (i.e., 500 and 750 μg/ml related to the particle concentration, *n* = 6) for 4 h at 37 and 4°C, respectively. Cells were washed with PBS, detached with 0.25% trypsin-EDTA and centrifuged at 3,000 rpm for 15 min (Centrifuge 5804 R, Eppendorf Austria GmbH, Vienna, Austria) at room temperature. The supernatant was removed and replaced with acetonitrile to disrupt cells and dissolve oil red o of the labeled particles. After sonification using EMAG Emmi®-D100 (EMAG AG, Mörfelden-Walldorf, Germany) for 10 min, cellular components were separated through centrifugation at 3,000 rpm for 15 min at room temperature. The absorbance of the supernatant was measured at a wavelength of 512 nm using the CLARIOstarPlus. Sample quantification was carried out by measuring the absorbance values in relation to those of the standards. For the preparation of the standards, labeled NP dissolved in and diluted with acetonitrile at different concentrations (i.e., 10–1,000 μg/ml) were used. The amount of internalized particles was calculated relative to the applied particle concentration (i.e., 500 and 750 μg/ml).

In parallel, inhibitors that selectively inhibit active transport pathways were used to identify the precise uptake mechanism (i.e., caveolin- or clathrin-mediated, macropinocytosis). For this, TR 146 cells were seeded in 24-well plates (glass bottom, Porvair Sciences, Wrexham, United Kingdom) with a seeding density of 4 × 10^4^ cells/well and incubated for 1 week. Prior, concentrations that did not induce harmful effects to the cells were evaluated for each inhibitor. Subsequently, TR 146 cells were pretreated with dynasore (i.e., 200 µM), chlorpromazine (i.e., 20 µM), genistein (i.e., 300 µM), and EIPA (i.e., 0.5 µM) for 40 min at 37°C respectively before incubation with the labeled SLN and NLC (i.e., 500 μg/ml) for 4 h at 37°C. (Tetyczka et al., 2021). After 4 h, particles were removed and cells were washed with PBS. Cytoskeleton was stained with 1 U Alexa Fluor 488 Phalloidin and nuclei were counterstained with 24 μg/ml Hoechst 33,342. Visualization of the particle uptake was performed *via* fluorescence microscopy using a confocal laser scanning microscope LMS 510 Meta (cLSM; Carl Zeiss GmbH, Vienna, Austria) equipped with a ZEN2008 software package. Labeled NP were detected at 633 nm excitation wavelength using a LP 650 nm long pass detection for the red channel. The cytoskeleton stained with Alexa Fluor 488 phalloidin was detected at an excitation wavelength of 488 nm using a BP bandpass of 505–550 nm for the green channel. Hoechst-stained nuclei were visualized at 505 nm excitation wavelength with a BP 420–480 nm bandpass detection for the blue channel. Images of randomly chosen areas of the cell monolayers were captured with cLSM. Z-stacks were acquired and virtual radial sections were documented.

For intracellular localization studies, TR 146 cells were seeded in 8-well chamber slides (ibidi GmbH, Martinsried, Germany) with a seeding density of 2 × 10^4^ cells/well and incubated for 2–3 days. Before incubation with labeled NP for 4 h at 37°C medium was removed and cells were washed with PBS. After 4 h, particles were removed and cells were washed with PBS. For staining of the cell organelles (i.e., mitochondria, endoplasmic reticulum and lysosomes), 100 nM Mito Red (Sigma Aldrich, Munich, Germany), 1 μMER Tracker Red (Thermo Fisher Scientific, Vienna, Austria) and 50 nM Lyso Tracker® Red DND-99 (Thermo Fisher Scientific, Vienna, Austria) were applied and incubated for 30 min. After that, cell nuclei were counterstained with Hoechst 33,342 and incubated for 10 min. Visualization was performed using a Nikon Eclipse Ti2 Microscope equipped with Andor Zyla sCMOS camera. Hoechst-stained nuclei were visualized at 395 nm excitation wavelength and 414–450 nm emission wavelength. Cell organelles were detected at 555 nm excitation wavelength and 580–611 nm emission wavelength and were shown in green. Imaging of the particles was performed at 640 nm excitation wavelength and 660–850 nm emission wavelength. Data processing was performed using NIS-Elements 5.21.03 software.

#### 2.2.4 Statistical Analysis

As not otherwise stated, experiments were performed in triplicate and the results were presented as mean values ±SD. Statistical analysis was performed using student’s t-test and differences were considered significant at a level of *p* ≤ 0.05 (*), *p* ≤ 0.01 (**), and *p* ≤ 0.001 (***).

## 3 Results

### 3.1 SLN and NLC Preparation and Characterization

**FIGURE 1:**
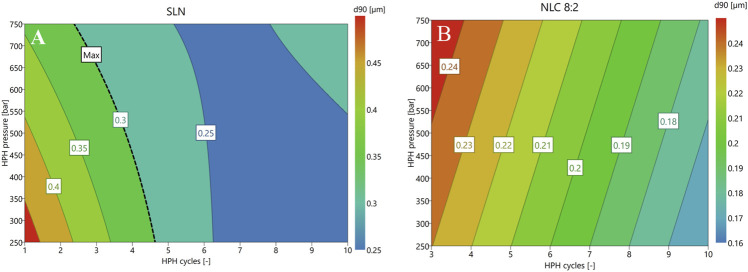
Response contour plots of SLN **(A)** and NLC formulations [**(B)**: 8:2; exemplary] at a fixed Tween 80 concentration of 2.5% (w/w) considering D (0.9) as response criterion. To simplify the model, small and insignificant interaction terms were excluded.

The DSC measurements showed that palmitic acid has a melting peak at 70.10 ± 0.20°C (with the onset at 62.30 ± 0.05°C) and oleic acid at 14.65 ± 0.05°C, which is both in accordance with the literature. (Knothe and Dunn, 2009; Kabri et al., 2011; Tetyczka et al., 2017; Kallakunta et al., 2018). Hence, the temperatures used for the HSM and HPH process were set 10°C above the melting temperature, i.e., at 71.5°C and the experimental runs of the DoE were performed as suggested by MODDE^®^. In this regard, for SLN production, coefficients plot identified the applied number of cycles and its quadratic effect as the most significant terms of the model. The same was observed for the NLC formulations. In addition, the matrix composition (i.e., lipid ratio) as well as its quadratic effect also proved to have a significant influence on the production of small particles with narrow particle size distribution (i.e., low span-value). The summary of fit for SLN and NLC showed high R2-values (i.e., 0.895 and 0.922), which indicate high fit between data and model and high Q2-values (i.e., 0.708 and 0.821), which represent reliability of the prediction. Moreover, via performing center point experiments in triplicate, reproducibility of the models was proven to be high (i.e., 1). Therefore, 2D contour plots were used to identify required number of cycles to achieve smallest particles with narrow particle size distribution (e.g., low span-value and/or low PdI) (see Figure 1). Accordingly, SLN and NLC particles were prepared applying 500 bar for six cycles.

**FIGURE 2:**
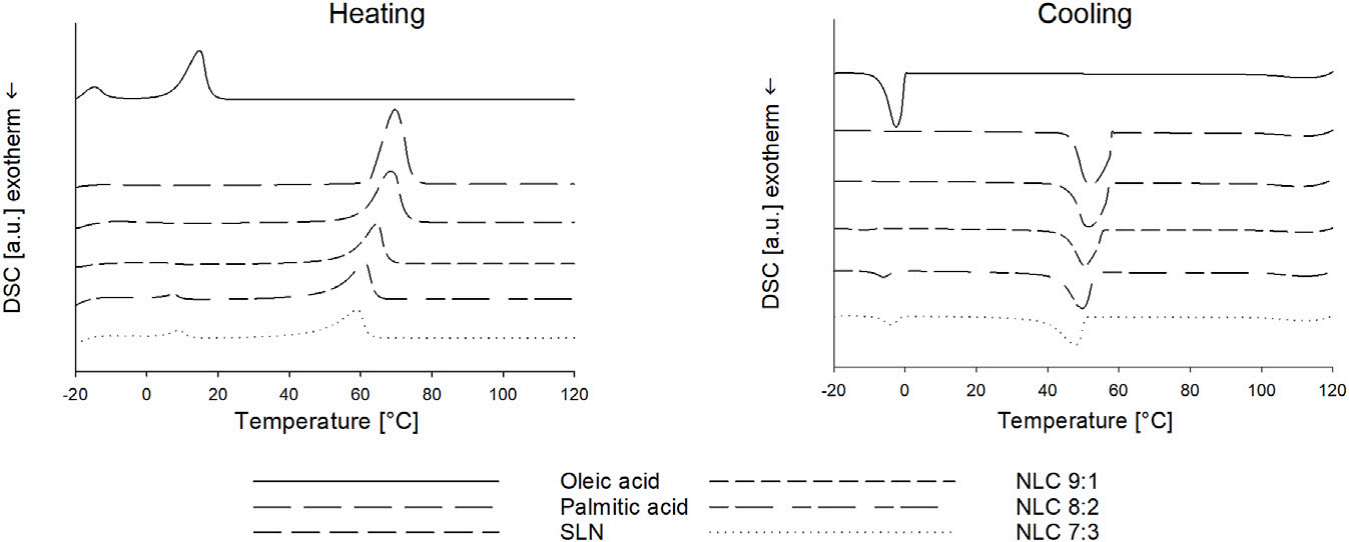
DSC thermograms of bulk palmitic acid and oleic acid and NP formulations (i.e., SLN and NLC).

After production, air-dried particles were investigated with regard to their thermal behavior. The SLN formulation exhibited a melting peak at 68.65 ± 0.25°C, which coincides with the bulk-lipid (see Figure 2). The DSC measurements of the NLC formulations showed that the melting peak of palmitic acid shifted to lower temperatures (i.e., 64.52 ± 0.32°C (NLC 9:1); 61.10 ± 2.78°C (NLC 8:2); 59.00 ± 0.67°C (NLC 7:3)) with increasing amount of liquid lipid. Moreover, the melting peaks of oleic acid were at 4.40 ± 0.05°C (NLC 9:1), 7.50 ± 0.10°C (NLC 8:2) and 9.00 ± 0.60°C (NLC 7:3) and thus increased with increasing liquid lipid amount. A significant increase in the melting enthalpy of oleic acid (i.e., 0.658 J/g (NLC 9:1), 6.751 J/g (NLC 8:2) and 12.520 J/g (NLC 7:3)) was observed, while the melting enthalpy of palmitic acid (i.e., 146.4 J/g (NLC 9:1); 149.0 J/g (NLC 8:2) and 116.2 J/g (NLC 7:3)) decreased as a function of oleic acid concentration. This suggests that the solid lipid has a crystalline nature and it can be assumed that polymorph C is dominating (see Figure 2). (Kelidari et al., 2017; Zoubari et al., 2017; Tetyczka et al., 2019; Kumar et al., 2020).

DSC can also provide information about the present NLC type and required cooling temperatures for the final nanoparticle formation. For palmitic acid and SLN recrystallization occurred at 52.00 ± 0.20°C and 51.54 ± 0.41°C, which slightly shifted towards lower temperatures with the addition of oleic acid, i.e., 50.60 ± 0.32°C (NLC 9:1), 49.70 ± 0.41°C (NLC 8:2) and 47.8 ± 0.64°C (NLC 7:3). The recrystallization enthalpies of palmitic acid (bulk = −243.4 J/g) also decreased with presence as SLN NP (i.e., −203.6 J/g) as well as with increasing oleic acid concentration (i.e., −147.8 J/g (NLC 9:1); −144.1 J/g (NLC 8:2); −114.2 J/g (NLC 7:3)) suggesting that oleic acid depressed not only the melting point but also crystallization temperature. The literature describes this as an indication that the formation of the less stable polymorphic form (i.e., B) is prevented and thus more stable imperfect and/or multiple-type of NLC are formed. (Tetyczka et al., 2019). In related studies performed by our group, it was also found that based on recrystallization behavior of the mixtures optimized cooling conditions for the final cooling process and adapted storage conditions are required to achieve high product quality (i.e., small sizes and narrow particle size distribution) and stability (i.e., prevention of gelling out). Here, it was found that both SLN and NLC required fast cooling and storage at 4°C to result in the desired product quality and stability.

Particle size was determined by DLS and LD (see Table 1). Immediately after preparation, the SLN formulations, diluted in MQ-water, had a Z-average value of 253.27 ± 2.23 nm and a PdI of 0.24 ± 0.01. The zeta potential was −28.33 ± 0.47 mV, which indicates that the particles are physically stable (at least over the measured time). (Mohapatra et al., 2018). The same observations were made for the NLC formulations. The Z-average values were 298.13 ± 3.08, 346.47 ± 3.69 and 363.87 ± 4.46 nm for 9:1, 8:2 and 7:3 ratios, respectively. The PdI values, which ranged from 0.24 ± 0.01 to 0.28 ± 0.03, show a narrow size distribution. This observation can be complemented by the LD measurements, more precisely, by the D (0.9) and D (0.99) values. Since the D (0.9) value below 250 nm is independent of the lipid matrix composition, it can be assumed that the main particle fraction does not increase in size as a function of liquid lipid amount. However, up to 10% larger particles and/or agglomerates (<0.7 µm) were formed due to the addition of oleic acid to the matrix. The zeta potential values were slightly higher (i.e., −32,57 ± 0.93 mV for 9:1, −31.40 ± 0.10 mV for 8:2 and −34.03 ± 0.25 mV for 7:3), suggesting appropriate physical stability.

**TABLE 1:** Particle characterization measurements including size (Z-average [nm]), PdI and ZP [mV] conducted via DLS and ELS. Volume based particle size classification performed via LD (i.e., D (0.1), D (0.5), D (0.9) and D (0.99) [µm].

	DLS/ELS			LD			
Sample	Z-Average [nm]	PdI	ZP [mV]	D (0.1) [µm]	D (0.5) [µm]	D (0.9) [µm]	D (0.99) [µm]
SLN	253.27 ± 2.23	0.24 ± 0.01	−28.33 ± 0.47	0.106 ± 0.002	0.170 ± 0.009	0.281 ± 0.003	0.480 ± 0.006
NLC 9:1	298.13 ± 3.08	0.26 ± 0.02	−32.57 ± 0.93	0.090 ± 0.004	0.161 ± 0.009	0.253 ± 0.008	0.630 ± 0.009
NLC 8:2	346.47 ± 3.69	0.24 ± 0.01	−31.40 ± 0.10	0.087 ± 0.005	0.148 ± 0.007	0.242 ± 0.008	0.664 ± 0.010
NLC 7:3	363.87 ± 4.46	0.28 ± 0.03	−34.03 ± 0.25	0.089 ± 0.003	0.148 ± 0.001	0.236 ± 0.002	0.662 ± 0.007

Stability studies were conducted for 1 month. The zeta potential values did not change during storage (i.e., > −30 mV); Z-average (<100 nm) and PdI values (<0.5) slightly increased. The supplemental LD measurements showed that the D (0.9) and D (0.99) of all NLC formulations slightly decreased to ∼200 and ∼220 nm during storage, which indicates that less than 1% larger particles were present. By contrast D (0.9) and D (0.99) of SLN increased in size to ∼600 and ∼1,450 nm. This is consistent with the volume-based particle size distribution, where the main fraction of SLN and NLC particles was in the constant size, however, a small peak was visible at larger sizes. To ensure that only deagglomerated nanostructures were used for the *in-vitro* experiments, freshly prepared SLN and NLC formulations were used for all experiments.

**FIGURE 3:**
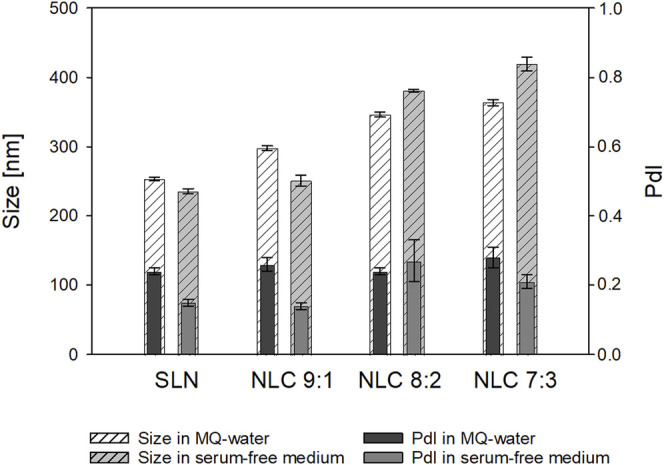
Mean-Z-average ± SD [nm] of SLN and NLC formulations dispersed in MQ-water and serum-free medium.

For the *in-vitro* studies, the stability of the nanostructures in cell culture medium (i.e., serum-free DMEM) was examined. It was found that no agglomeration occurred (see Figure 3).

**FIGURE 4:**
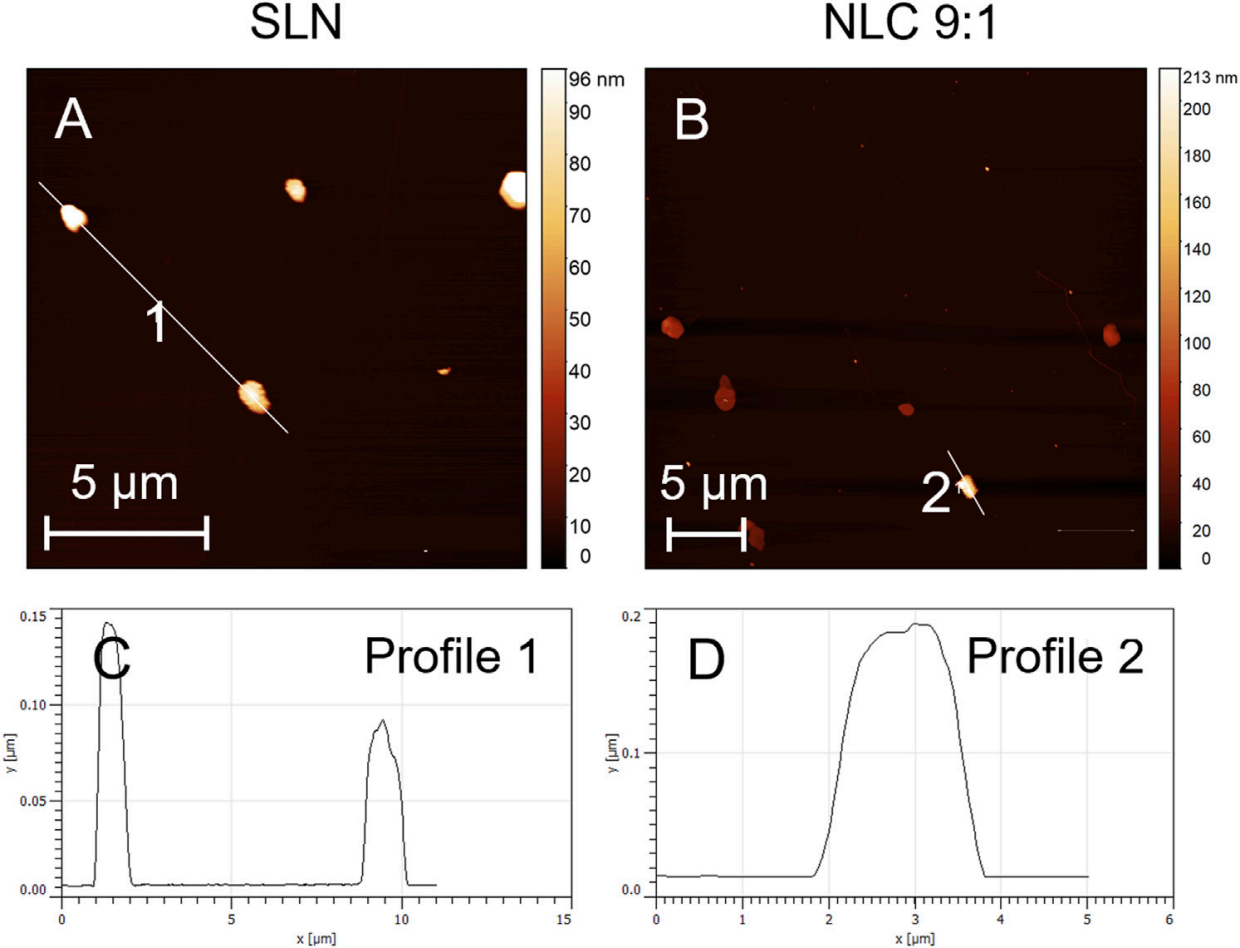
Topographical AFM images of SLN **(A)** and NLC using the 9:1 formulation as an example **(B)** and the corresponding surface height profiles i.e., **(C)** (SLN) and **(D)** (NLC 9:1).

AFM images, which were performed to determine the particle shape are shown in Figure 4. Topographical imaging of SLN and NLC (Figures 4A,B) including a surface height profile (Figures 4C,D) revealed spherical shaped particles with sizes between 80 and 170 nm, which coincide with measured D (0.1) and D (0.5) values of LD measurements.

### 3.2 Cytotoxicity and Uptake Studies

#### 3.2.1 Cytotoxicity Studies

**FIGURE 5:**
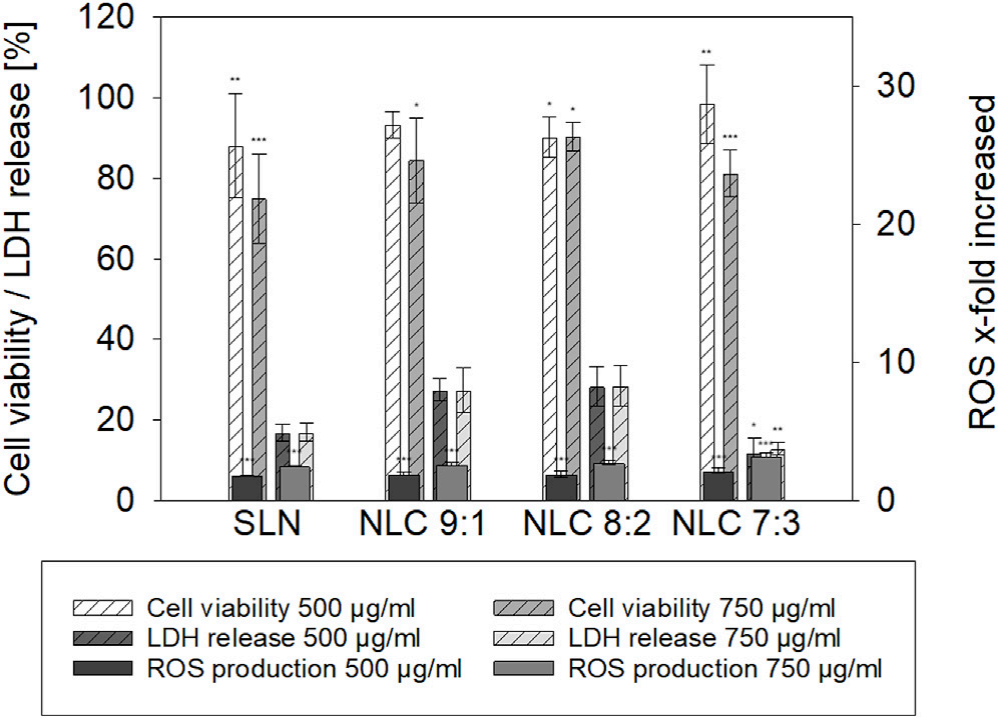
Cell viability [%], LDH release [%] and ROS production [x-fold increase compared to cell control] of TR 146 treated with palmitic acid formulations. The percentages shown (% ± SD) refer to the corresponding controls. Statistical analysis was performed using student’s t-test and significant differences compared to the control are marked as * (*p* value <0.05), ** (*p* value <0.01) and *** (*p* value <0.001) respectively.

Cell viability of TR 146 cells was studied after incubation with SLN and NLC formulations in a dose-dependent manner (i.e., 500 and 750 μg/ml) by quantifying the metabolic activity *via* MTS tests (see Figure 5). It was observed that the viability of the cells slightly decreased with increasing particle concentration but was still above 70% for SLN and higher than 80% for NLC. (Teubl, Meindl, et al., 2013). This correlates with the LDH tests (see Figure 5). For all formulations tested, the LDH release was below 30% compared to the cell control indicating no detrimental effects on the cell membrane.

Oxidative stress responses of 500 and 750 μg/ml SLN and NLC formulations are also shown in Figure 5. Incubation of TR 146 cells with SLN increased ROS release 1.8-fold and 2.5-fold at 500 and 750 μg/ml particle concentration, respectively, compared to the untreated cell control. Increasing amounts of oleic acid in the NLC formulations also resulted in a significant increase of ROS release in a dose-dependent manner. At lower particle concentrations, ROS-release increased from 1.9-fold to 2.1-fold and was further increased from 2.6-fold to 2.7- and 3.2-fold when testing 750 μg/ml of 9:1, 8:2 and 7:3 lipid ratios.

#### 3.2.2 Uptake Studies

**FIGURE 6:**
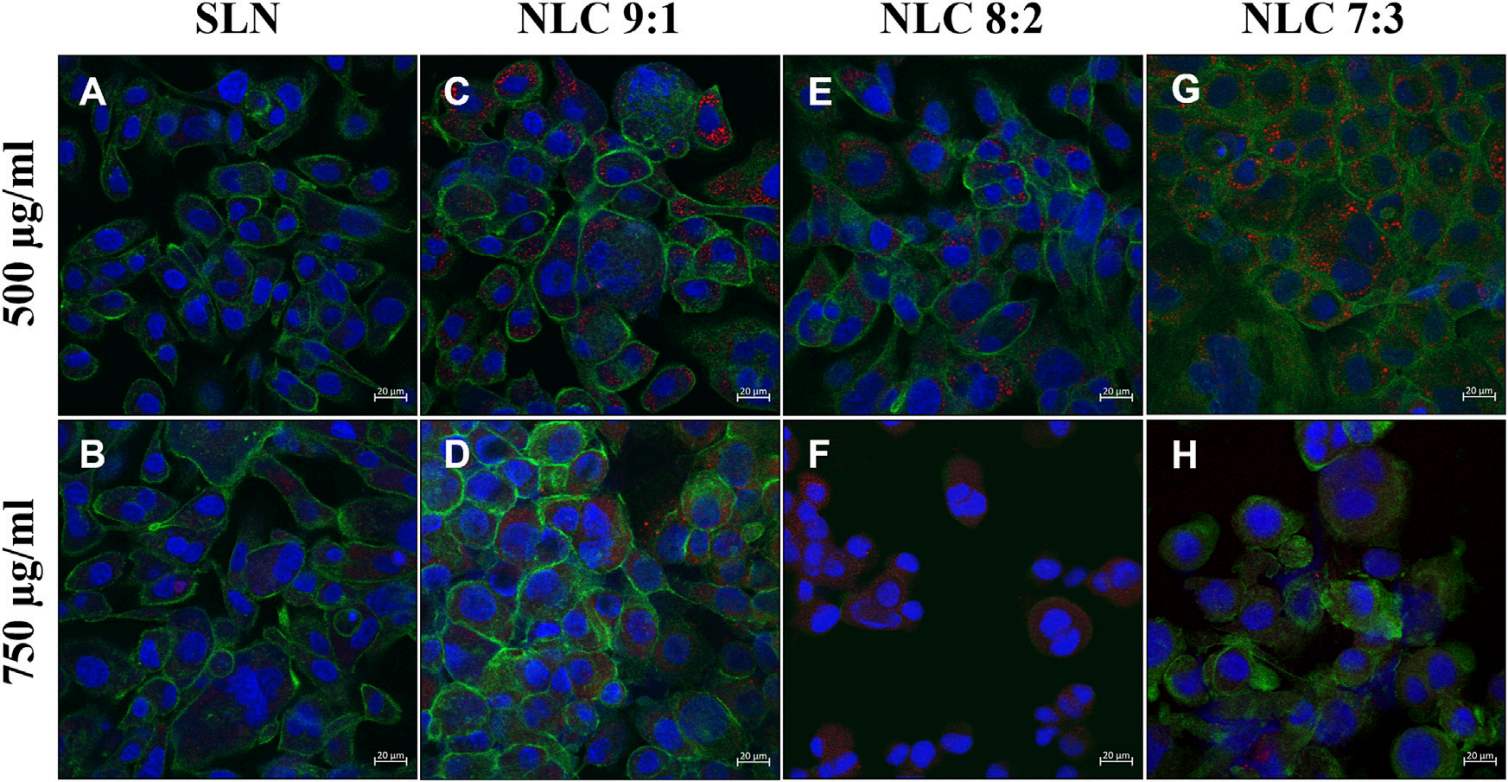
TR 146 cells after incubation with 500 μg/ml and 750 μg/ml SLN **(A,B)** and 500 μg/ml and 750 μg/ml NLC particles (i.e., 9:1 **(C,D)**, 8:2 **(E,F)** and 7:3 **(G,H)**). The cytoskeleton was stained with Alexa Fluor 488 Phalloidin (green) and nuclei were counterstained with Hoechst 33,342 (blue). NP were labeled with oil red o (red).

**FIGURE 7:**
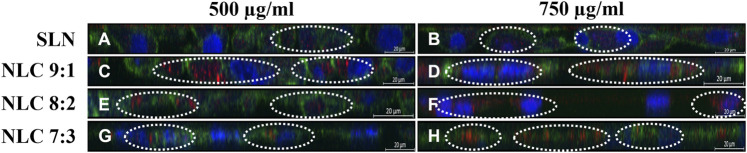
cLSM z-scans of TR 146 cells after incubation with 500 μg/ml and 750 μg/ml SLN **(A,B)** and 500 μg/ml and 750 μg/ml NLC particles (i.e., 9:1 **(C,D)**, 8:2 **(E,F)** and 7:3 **(G,H)**). The cytoskeleton was stained with Alexa Fluor 488 Phalloidin (green) and nuclei were counterstained with Hoechst 33,342 (blue). NP were labeled with oil red o (red).

Cellular particle uptake was investigated in a dose dependent manner and the effect of matrix composition on the uptake efficacy was evaluated. It was found that the SLN formulations did not exert any obvious stress to the cells (see Figures 6A,B, 7A,B). These observations coincide with the semi-quantitative studies, which showed that at low and high concentrations only a few isolated particles were detected inside the cells (particle uptake less than 4%). In contrast, the addition of oleic acid to the lipid matrix resulted in a markedly enhanced particle uptake. At concentrations of 500 μg/ml approximately 5% NLC (9:1, 8:2 and 7:3 ratios) penetrated the cells, at higher concentrations (750 μg/ml) up to 14% were found inside the cells. More specifically, when the proportion of oleic acid in the matrix was increased from 9:1 to 8:2 or 7:3, the following observations were made: when 500 μg/ml of the particles were applied to the cells, the morphological structure of the cells was not affected in terms of cytoskeletal structure, as shown in Figures 6C,E,G (see also Figures 7C,E,G). Further increase of the applied concentration to 750 μg/ml (see Figures 6D, **F, H**, 7D, **F,H**) did not influence the performance of the 9:1 formulation (see Figure 6D). However, incubation with the 8:2 and 7:3 formulations resulted in damage of the intact cytoskeletal structures (see Figures 6F,H) which coincides with the cytotoxicity studies.

**FIGURE 8:**
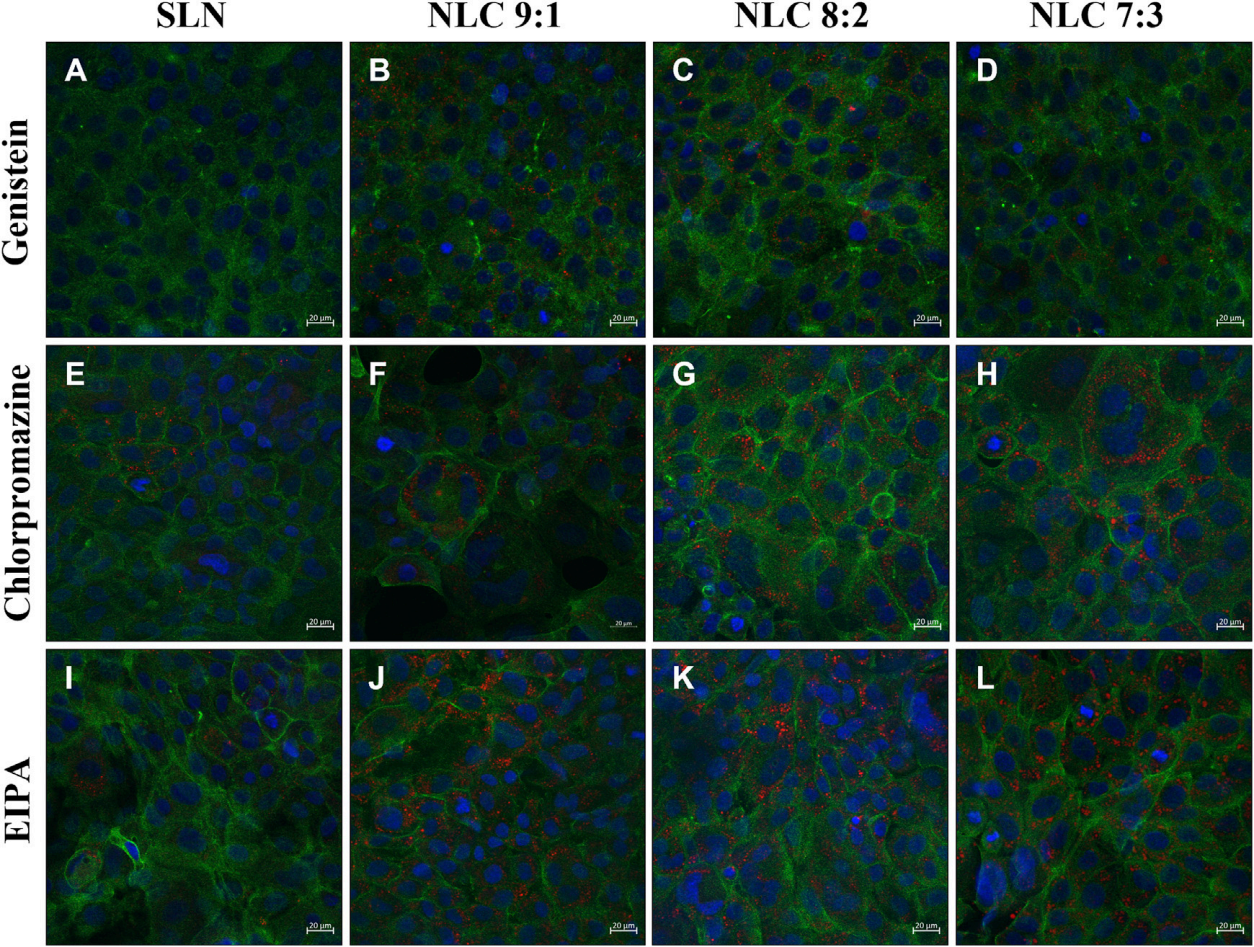
Localization of particles in TR 146 cells after pretreatment with genistein **(A–D)**, chlorpromazine **(E–H)** and EIPA (I–L) before incubation with 500 μg/ml SLN **(A,E,I)** and 500 μg/ml NLC particles (i.e., 9:1 **(B,F,J)**, 8:2 **(C,G,K)** and 7:3 **(D,H,L)**). The cytoskeleton was stained with Alexa Fluor 488 Phalloidin (green) and nuclei were counterstained with Hoechst 33,342 (blue). NP were labeled with oil red o (red).

**FIGURE 9:**
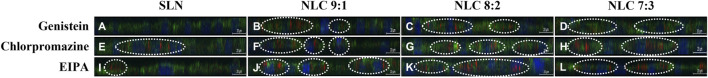
cLSM z-scans of TR 146 cells after pretreatment with genistein **(A–D)**, chlorpromazine **(E–H)** and EIPA (I–L) before incubation with 500 μg/ml SLN **(A,E,I)** and 500 μg/ml NLC particles (i.e., 9:1 **(B,F,J)**, 8:2 **(C,G,K)** and 7:3 **(D,H,L)**). Cell nuclei were stained with Hoechst 33,342 (blue) and the cytoskeleton was counterstained with Alexa Fluor 488 Phalloidin (green). NP were labeled with oil red o (red).

The uptake pathway, i.e., active versus passive and the respective routes were investigated using particle concentrations of 500 μg/ml and inhibitor concentrations that did not affect cell morphology. (Tetyczka et al., 2021). Cells incubated at 4°C with 500 μg/ml particles showed no uptake regardless of the formulation tested, hence passive uptake could be excluded (data not shown). Incubating the cells at 37°C with dynasore to inhibit caveolin- and clathrin-mediated endocytosis, resulted in no particle uptake (data not shown). Specific inhibition of the caveolin-mediated route by genistein revealed that a small amount of NLC could be detected inside the cells (see Figures 8, 9B–D); by contrast, SLN were not internalized (see Figures 8A, 9A). Inhibition of the clathrin-mediated route by chlorpromazine resulted in a minor uptake of SLN (see Figures 8E, 9E) and an increased internalization of the NLC (see Figures 8F–H, 9F–H). Since a considerable number of particles could be visualized in the cell even after inhibition of macropinocytosis with EIPA (see Figures 8I–L, 9I–L), it can be concluded that caveolin-mediated endocytosis represents the main uptake pathway route for SLN and NLC particles. However, clathrin-mediated endocytosis was only marginally involved in the uptake of NLC particles into the cell.

**FIGURE 10:**
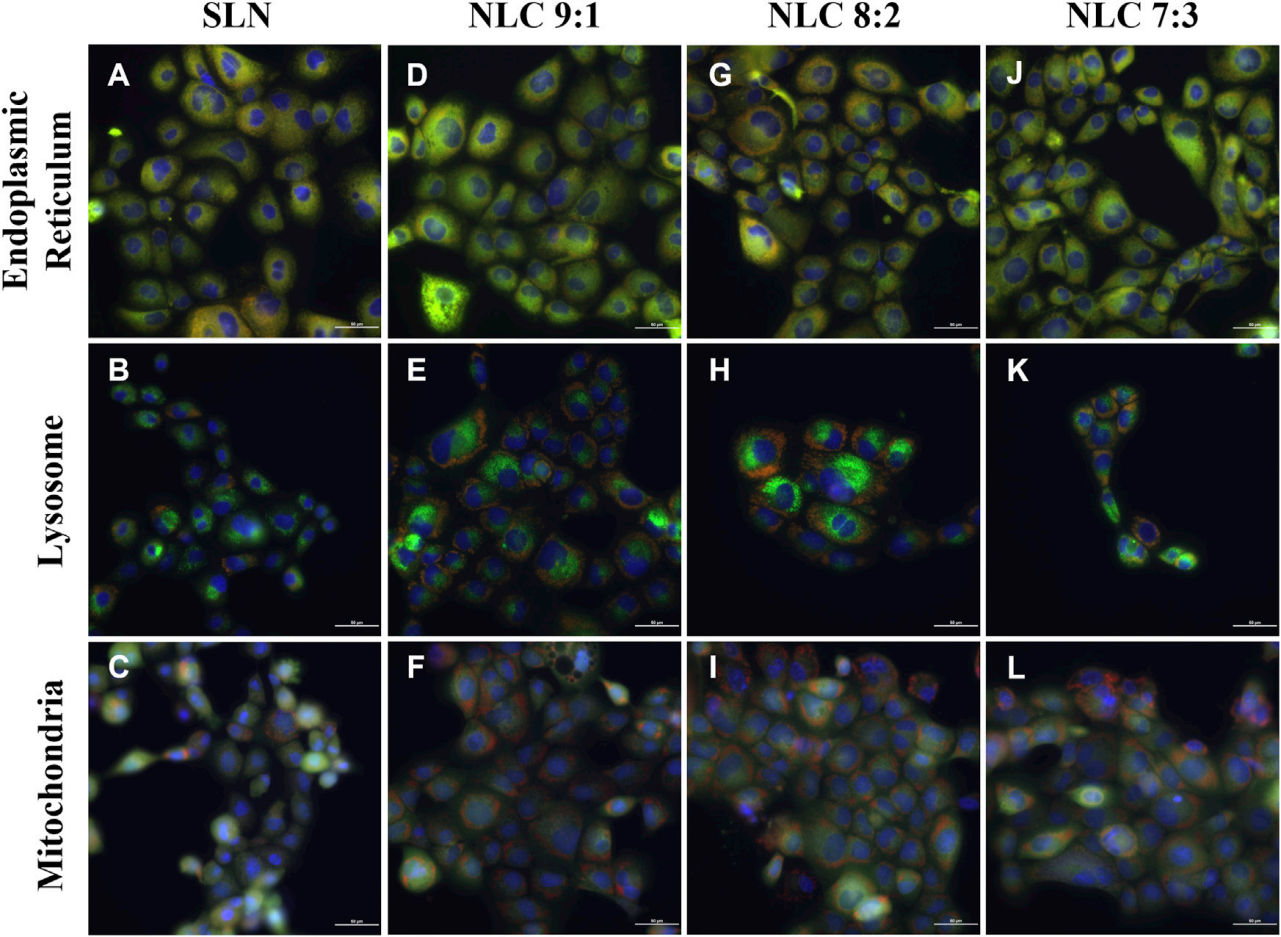
Visualization of intracellular localization of 500 μg/ml labeled NP (red) (SLN **(A–C)** and NLC (9:1 **(D–F)**, 8:2 **(G–I)** and 7:3 **(J–L)** after incubation for 4 h at 37°C. Endoplasmic reticulum **(A–J)**, lysosomes **(B–K)** Mitochondria **(C–L)** are visualized in green. Cell nuclei were stained with Hoechst 33,342 (blue).

Intracellular localization studies showed that regardless of the matrix composition, most particles could be found in the endoplasmic reticulum (see Figures 10A,D,G,J). In addition, SLN and NLC particles were not located in the lysosomes but at their periphery (see Figures 10B,E,H,K) and only a few particles could be detected near the mitochondria (see Figures 10C,F,I,L). This is consistent with the observed uptake mechanism, as SLN and NLC entered the cells mainly by caveolin-dependent endocytosis. However, NLC particles with the highest oleic acid content (i.e., 7:3) were partially localized in lysosomes (see Figure 10K).

## 4 Discussion

Nano-drug delivery systems (DDS) must penetrate cells safely and effectively to reach intracellular targets and to achieve therapeutic efficacy. Hence, tuning of the physicochemical properties of NP is considered crucial taking into account the anatomical and physiological characteristics of the target tissue or organ. (Behzadi et al., 2017; Foroozandeh and Aziz, 2018). The rational design of such a carrier system starts with the selection of an appropriate lipid matrix composition (considering the drug´s properties), followed by suitable manufacturing processes that allow reproducible production of nanosystems with defined product properties. Together with careful characterization, this is a prerequisite to be able to perform reliable biological *in-vitro* tests to assess efficiency and intracellular fate.

To achieve fabrication of SLN and NLC, HSM and HPH were used. The DoE studies showed that both the formulation composition (lipid ratios) and number of applied cycles show significant effect on the resulting SLN and NLC particle size. The statistical analysis of the performed experimental studies predicted high fit between data and model and high reliability of prediction, since R2 and Q2 were >0.5 and differences between R2 and Q2 were <0.3. (Satorius Stedim Data Analytics AB, 2017). Adjustment of the process and formulation parameters according to the contour plots enabled the reproducible production of particles with a size of about 250 nm, a low PdI (<0.3) and a negative surface charge. More precisely, the D (0.9) values of SLN and NLC particles after preparation revealed sizes ≤280 nm. Since the actual sizes fitted well with the predicted values, DoE studies can be considered a valuable tool for reproducible manufacturing of nanosystems with desired properties. In addition, the zeta potential values were negative and did not change during storage, resulting in almost stable systems. The AFM images show that the sizes visualized are consistent with the sizes measured. Moreover, they suggest that SLN and NLC particles are spherically shaped which coincides with studies performed by Tetyczka et al. (Tetyczka et al., 2019) and Rakotoarisoa et al. (Rakotoarisoa et al., 2021) using cryo-transmission electron microscopy combined with synchrotron small-angle X-ray scattering. The shifts in the thermal profiles of the particles allowed identifying the type of lipid system present. The SLN formulations showed only a slight shift of the endotherm towards lower temperatures. This can be attributed to the presence of surfactant domains and smaller particle sizes of the powder and is a phenomenon frequently described in the literature. (Kumar et al., 2020). Accordingly, classic SLN formulations that were solid and crystalline at room temperature were present. These data are in accordance with the literature. (Arana et al., 2015; Kumar et al., 2020). Compared to SLN, which show several disadvantages including random gelation tendency, morphological changes resulting in drug leakage/expulsion during storage, and a poor loading capacity, NLC can overcome these limitations. (Müller, Radtke and Wissing, 2002a). The thermograms (see Figure 2) of the NLC formulations showed that the melting point of palmitic acid shifted to lower temperatures due to the addition of oleic acid. Thereby, larger distances between the fatty acid chains of the solid lipid matrix were formed exhibiting a highly disordered matrix, which provides free spaces for drug accommodation. (Müller, Radtke and Wissing, 2002a; Kovačević, et al., 2020). At the 9:1 ratio, the crystalline nature of the solid lipid was also found to decrease, indicating the formation of an imperfect type. (Müller, Radtke and Wissing, 2002a; Pardeike, Hommoss and Müller, 2009; Beloqui et al., 2016; Tetyczka et al., 2017, 2019; Chauhan et al., 2020). The corresponding melting peak of oleic acid also shifted to lower temperatures and increased in intensity with increasing amount of liquid lipid. This indicates that at ratios of 8:2 and 7:3, the multiple-type of NLC was formed. The multiple-type of NLC is characterized by liquid oily nano-compartments in the solid lipid matrix, in which consequently poorly soluble drugs can be dissolved to a high extent. (Müller, Radtke and Wissing, 2002b; Üner and Yener, 2007). The formation of oily nano-compartments results from phase separation through miscibility gaps leading to precipitation during the cooling process. These small liquid lipid droplets were visible as crystallization peaks in the DSC thermograms (see Figure 2).

Under physiological conditions, particles first encounter saliva prior to interacting with the underlying epithelium. Saliva is a hypotonic fluid containing proteins, enzymes and inorganic salts such as calcium, phosphate, (bi)carbonate and others. (Teubl et al., 2018). They partly act as buffer to keep the pH value in the mouth in the neutral range. (Marsh et al., 2016). Thereby, salivary mono- and divalent cations may also reduce the thickness of the diffuse layer formed close to a NP surface, which in turn can lead to an enhanced agglomeration tendency and therefore instability. (Moore et al., 2015). In our study, neither monovalent nor divalent inorganic ions were found to alter the size or PdI of both SLN and NLC. These observations agree well with Teubl et al. (Teubl, Absenger, et al., 2013) who showed that negatively charged polymer particles in saliva show less interaction and agglomeration than positively charged particles. Since agglomeration of the particles could be excluded, the particles were incubated with TR 146 cells. SLN showed minor cell uptake. These data also correlate with the cytotoxicity assays. In addition, no effects on the cell morphology, e.g., formation of stress fibers, disruption of the cytoskeletal network and influence on membrane integrity were observed. By contrast, with the addition of oleic acid, particle uptake into TR 146 cells was enhanced. This emphasizes, that particle uptake into buccal epithelial cells is not only related to size and charge but also to the lipid composition. The addition of the unsaturated fatty acid facilitated particle-membrane interaction and thus uptake, which coincides with Foroozandeh et al. (Foroozandeh and Aziz, 2018). Thereby, oleic acid, which is also a known penetration enhancer, increased the fluidization of the lipid cell membrane. (Ibrahim and Li, 2010; Mendonsa et al., 2017; Hansen et al., 2018). However, the microscopic investigations and the cytotoxicity studies revealed that (morphological) changes occurred with higher particle concentrations and increasing amounts of oleic acid. Treatment of cells with 8:2 and 7:3 NLC formulations at higher particle concentrations (i.e., 750 μg/ml) showed cell stress responses (Figures 5F, H) and resulted in a significant (*p* < 0.01 and 0.001) reduction in cell viability. As described in the literature, both the endocytosis and exocytosis profiles of NP strongly depend on the applied doses, which complies with the findings of our studies, as higher doses led to higher uptake capacity. However, the size of the NP determines their delivery into fast or slow recycling compartments. Thereby, larger particles are more likely to be located in the slow recycling compartments, which further slows down exocytosis. (Sakhtianchi et al., 2013). This leads to an accumulation of NP inside the cell, which influences cell viability. The reason for this is that oleic acid affects the permeabilization of the outer and/or inner membrane of mitochondria, leading to the release of pro-apoptotic intermembrane proteins (e.g. apoptosis-inducing factor). (Di Paola and Lorusso, 2006). In this process, free fatty acids increase the proton conductance of the inner membrane by acting as a protonophore. This increases the permeability of the inner membrane of Ca^2+^-loaded mitochondria, leading to compensation of small solutes across the membrane. This, in turn, causes matrix proteins to generate osmotic pressure leading to matrix swelling, followed by rupture of the outer membrane and subsequent release of pro-apoptotic intermembrane proteins. (Di Paola and Lorusso, 2006). These results are consistent with the findings on ROS formation. Increasing proportions of oleic acid led to significantly increased ROS production in a concentration-dependent manner (i.e., 3.3-fold for 7:3 ratio at 750 μg/ml). Moreover, oleic acid has been shown to interact with the amphiphilic phospholipid cell membranes due to their structural properties (i.e., single-chain lipid amphiphiles with hydrocarbon chain and hydrophilic head group). Depending on the intensity of interaction, membrane defects such as solubilization, pore formation or shape change can be triggered. Such membrane interactions can be observed in LDH release studies and were reported to be considered beneficial for particle uptake when they do not affect cell viability. (Tan et al., 2021).

Our results showed that the effect of particle concentration on cell membrane integrity was not as pronounced as in the MTS studies. The amount of LDH released was similar to the cell control. In particular, all tested NLC formulations did not cause LDH release higher than 30%. Dependencies on matrix composition were also not clearly evident, with the exception of the 7:3 NLC formulations, which surprisingly caused significantly less damage. Regarding the exact uptake route involved, those NLC formulations that did not induce any adverse cell effects were taken up into the cells. Thereby, passive diffusion is predominantly limited to small, uncharged molecules that diffuse along a concentration gradient. (Foroozandeh and Aziz, 2018; Mitchell et al., 2021). Similarly, only NP with a size smaller than the thickness of the membrane bilayer (i.e., <60 nm) can passively enter the cytosol of living cells, which was not the case in our study. (Canepa et al., 2021). Here, SLN and NLC were exclusively taken up via active pathways. Thereby, Rivolta et al. reported that SLN utilize exclusively clathrin-mediated endocytosis pathways in alveolar epithelial cells. (Rivolta et al., 2011). By contrast, it is reported that organic negatively charged particles are preferentially internalized by caveolin- and/or clathrin-mediated endocytosis in human alveolar epithelial cells. (Brandenberger et al., 2010). Moreover, there is evidence that particles smaller than 200 nm are taken up by clathrin-coated pits, while larger structures are taken up by caveolin-mediated endocytosis in non-phagocytic B16 cells. (Rejman et al., 2004). This is consistent with our results showing that caveolin-mediated endocytosis is the major pathway and the clathrin-mediated pathway was only involved to a minor extent. As the inhibitory effect of the chemicals used is extremely cell type dependent, and the concentration that minimizes cellular toxicity while maximizing the inhibitory effect may influence the uptake pathway, co-localization studies were performed without prior incubation with chemical inhibitors. (Teubl, Meindl, et al., 2013; Schimpel et al., 2016). These studies confirmed caveolin-endocytosis as the main uptake pathway, as SLN as well as NLC were found to accumulate in the endoplasmic reticulum. (Kou et al., 2013).

## 5 Conclusion

Nano DDS with their multiple crucial properties including size, charge, shape, hydrophobicity, matrix material etc. offer an excellent strategy to treat oral mucosal disorders locally with complex drug candidates. The diverse properties, however, make it difficult to predict and control both safety and efficacy of nano DDS. This is because smallest changes in physicochemical properties, e.g., due to drug-specific formulation adaptations, can already trigger massive changes in biopharmaceutical performance. In this study, an in-depth knowledge of the biological interactions and safety of lipid drug carriers differing in matrix composition was built. Based on a predictive model obtained through DoE studies, SLN formulations and NLC formulations could be reproducibly prepared. Thereby, by adding different concentrations of oleic acid, different types of NLC, namely imperfect and multiple-type, could be produced, which allow variations in drug loading. All particles exhibited reasonable product quality in terms of size, particle size distribution and surface charge. The average size of all formulations was about 250 nm, the surface charge was negative and all particles showed a spherical shape, which underlines the importance of DoE studies and quality by design approaches as valuable tools for reproducible NP production. As an important prerequisite for effective particle uptake, no significant adverse influence of, for example, ions physiologically present in saliva on colloidal stability could be observed. Compared to SLN, oleic acid contained in the NLC formulations significantly increased cellular uptake capacity in a concentration-dependent manner. Regardless of the matrix composition, uptake occurred mainly via caveolin-mediated endocytosis, which was associated with localization of the particles in the endoplasmic reticulum. At the same time, higher liquid lipid levels induced concentration-dependent cytotoxic effects due to interactions with the mitochondrial membrane.

In summary, NLC are taken up by the oral mucosa at optimal particle size and surface charge. By adding different concentrations of oleic acid, different types of NLC can be prepared, thus broadening the range of applications as DDS. The matrix composition per se has an impact on the biopharmaceutical performance, especially in terms of internalization capacity, but not on the uptake pathway itself.

## Data Availability

The original contributions presented in the study are included in the article/Supplementary Material, further inquiries can be directed to the corresponding author.
